# Novel artificial intelligence-based hypodensity detection tool improves clinician identification of hypodensity on non-contrast computed tomography in stroke patients

**DOI:** 10.3389/fneur.2024.1359775

**Published:** 2024-02-15

**Authors:** Angela Dos Santos, Milanka Visser, Longting Lin, Andrew Bivard, Leonid Churilov, Mark William Parsons

**Affiliations:** ^1^University of New South Wales, South-Western Sydney Clinical Campus, Kensington, NSW, Australia; ^2^Faculty of Medicine, Dentistry and Health Sciences, University of Melbourne, Melbourne, VIC, Australia; ^3^Melbourne Brain Centre, University of Melbourne, Melbourne, VIC, Australia; ^4^Sydney Brain Centre, Faculty of Medicine, University of New South Wales, Kensington, NSW, Australia; ^5^Melbourne Medical School, University of Melbourne, Melbourne, VIC, Australia; ^6^Department of Neurology, Liverpool Hospital, Ingham Institute for Applied Medical Research Liverpool, Liverpool, NSW, Australia

**Keywords:** automated hypodensity detection tools, artificial intelligence, machine learning, acute ischaemic stroke, treatment

## Abstract

**Introduction:**

In acute stroke, identifying early changes (parenchymal hypodensity) on non-contrast CT (NCCT) can be challenging. We aimed to identify whether the accuracy of clinicians in detecting acute hypodensity in ischaemic stroke patients on a non-contrast CT is improved with the use of an Artificial Intelligence (AI) based, automated hypodensity detection algorithm (HDT) using MRI-DWI as the gold standard.

**Methods:**

The study employed a case-crossover within-clinician design, where 32 clinicians were tasked with identifying hypodensity lesions on NCCT scans for five *a priori* selected patient cases, before and after viewing the AI-based HDT. The DICE similarity coefficient (DICE score) was the primary measure of accuracy. Statistical analysis compared DICE scores with and without AI-based HDT using mixed-effects linear regression, with individual NCCT scans and clinicians as nested random effects.

**Results:**

The AI-based HDT had a mean DICE score of 0.62 for detecting hypodensity across all NCCT scans. Clinicians’ overall mean DICE score was 0.33 (SD 0.31) before AI-based HDT implementation and 0.40 (SD 0.27) after implementation. AI-based HDT use was associated with an increase of 0.07 (95% CI: 0.02–0.11, *p* = 0.003) in DICE score accounting for individual scan and clinician effects. For scans with small lesions, clinicians achieved a mean increase in DICE score of 0.08 (95% CI: 0.02, 0.13, *p* = 0.004) following AI-based HDT use. In a subgroup of 15 trainees, DICE score improved with AI-based HDT implementation [mean difference in DICE 0.09 (95% CI: 0.03, 0.14, *p* = 0.004)].

**Discussion:**

AI-based automated hypodensity detection has potential to enhance clinician accuracy of detecting hypodensity in acute stroke diagnosis, especially for smaller lesions, and notably for less experienced clinicians.

## Introduction

The mainstay of acute stroke imaging has been computed tomography (CT). It is relatively accessible in most hospitals throughout the world, is inexpensive compared with magnetic resonance imaging (MRI), efficient, fast and has few contraindications (1). However, in the first few hours after stroke onset, identification of the early signs of ischaemic stroke (parenchymal hypodensity and focal swelling) on non-contrast CT (NCCT) (1–3) can be challenging for even the most experienced clinicians (4). Image interpretation can delay therapeutic decisions and is often the rate limiting step, particularly if the radiologist is offsite, which often is the case in rural and remote Australia for example (5, 6). For the onsite clinicians, fatigue and inexperience can affect image interpretation and delay treatment decisions (1). However, identification of these subtle changes (particularly parenchymal hypodensity) is necessary as they likely represent irreversible ischaemia and this is an important consideration in the decision to offer reperfusion therapy (6).

As reperfusion treatment is time critical, decision support tools such as artificial intelligence (AI) based automated hypodensity detection have the potential to improve detection of early ischemic change and reduce delays in diagnosis and reperfusion treatment (1).

Current literature describes several approaches for hypodensity detection for NCCT images in stroke. For example, image filtering (windowing) to enhance the visibility of ischaemic changes (7, 8), spatial normalisation between a template of healthy controls and the examined brain (9, 10), topographic scoring using the territories of the middle cerebral artery (MCA) (11, 12), classification of the image texture features (13) and imaging biomarkers (14). A recent meta-analysis demonstrated that AI-driven tools had performed either comparable to or surpassed that of physicians in the assessment of early changes after stroke (15). This indicates that AI-based hypodensity detection tools have the potential to improve clinician performance, however this has not been assessed previously.

One AI-based automated NCCT hypodensity detection tool (HDT, MIstar, Apollo Medical Imaging, Melbourne, Australia) uses histogram-based left-right brain comparisons to detect regions-of-interest that show unilateral hypodense areas. It uses iterative level-set optimization to identify areas of hypodensity within a non-contrast CT scan. This AI-based HDT showed strong positive correlation with the gold standard, magnetic resonance imaging diffusion-weighted imaging (MRI-DWI) (correlation coefficient >0.5, unpublished data).

Thus, the aim of this study was to investigate whether the accuracy of clinicians in detecting acute hypodensity in ischaemic stroke patients on non-contrast CT is improved with the use of an AI-based automated HDT algorithm.

We hypothesized that the accuracy of clinicians in detecting acute hypodensity in ischaemic stroke patients on non-contrast CT will be improved with the use of an AI-based automated HDT algorithm.

## Methods

### Study design

**Figure 1:**
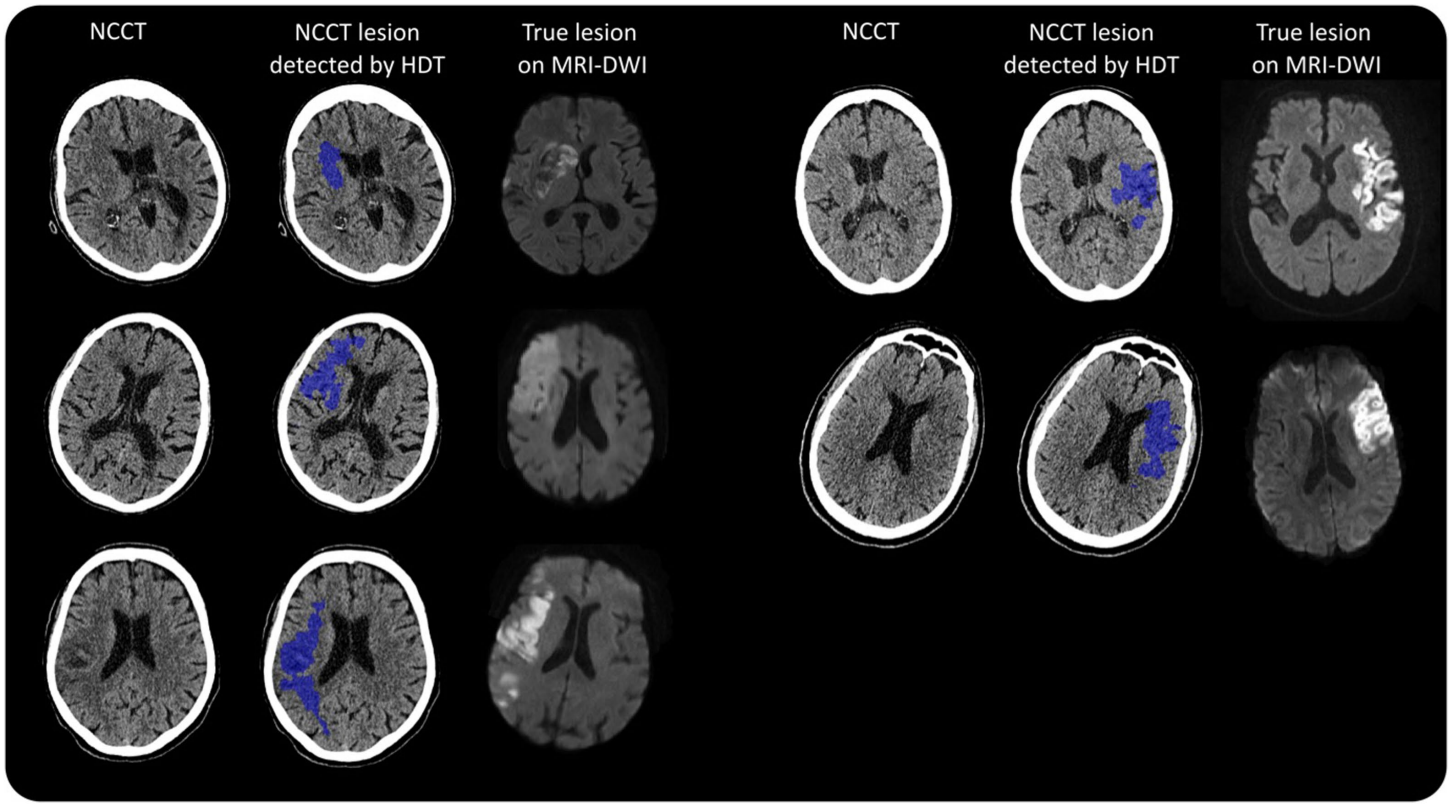
Case selection top left: Image ID 1, middle left: Image ID 2, bottom left: Image ID 3, top right: Image ID 4, middle right: Image ID 5.

This was a case-cross over within-clinician study where clinicians were asked to identify hypodensity lesions first before, and then after, the help of the AI-based HDT output on a set *a priori* purposively sampled patient cases that represent the broad population of acute stroke patients. While both individual clinicians and case scans can be considered as sources of variability in this design, the objective of investigating clinicians accuracy dictates the need to treat clinicians as units of analysis. Individual scans were purposively selected to represent variant lesion size and hemisphere, representative of acute stroke, of the anterior circulation. Among the 5 NCCT scans, 2 had large lesions with hypodensity involving more than 2/3 the MCA territory, and 3 scans had small lesions. All scans had a corresponding MRI-DWI during admission. The summary of selected case characteristics is presented in Figure 1 and Table 1.

**Table 1:** Case selection description.

Cases	Infarct description	Stroke onset time to CT in hours
1	Small right basal ganglia infarct	4.1
2	Small right frontal infarct	1.2
3	Large right fronto-temporal parietal infarct	5.7
4	Large left fronto-temporal infarct	10.1
5	Small left frontal infarct	7.9

**Figure 2:**
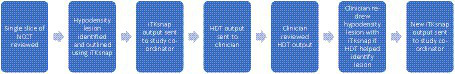
Schematic diagram of methods.

Clinicians were instructed to manually segment a single slice of five different patients’ NCCT scans before having access to the AI-based HDT output (Figure 2). Once they had segmented the single slice and sent their segmentation back to the study coordinator (MV), they were given the AI-based HDT output that had identified the hypodensity on the same single slice. The clinicians were asked to compare the AI-based HDT output and their initial drawing. They were asked to draw a new lesion if they felt the AI-based HDT helped identify the hypodensity. They then sent their completed segmentations back to the study coordinator for analysis.

### Power analysis

As the primary objective of this study was to investigate clinicians’ accuracy, power was calculated on the need to treat clinicians as units of analysis. Recruiting 32 clinicians provided 0.8 power to detect the medium effect size (Cohen’s *d* = 0.5) for the difference in means of DICE score (dependent samples/matched pairs without and with the use of AI-based HDT) under the settings of two-sided type I error of 0.05. The clinicians were either neurology trainees with less than 5 years experience reading NCCT in acute stroke patients or consultant neurologists with more than 5 years experience.

### Study scans

#### Automated NCCT AI-based hypodensity detection tool

The NCCT scans in this study were obtained from the Toshiba Aquilion One from two INternational Stroke Perfusion Imaging REgistry (INSPIRE) sites (Canon, Tokyo, Japan). The NCCT lesion was segmented automatically with the AI-based HDT algorithm on MIStar software as illustrated in Figure 1 (Apollo Medical Imaging, Melbourne, Australia). It consisted of the following steps: (1) assessing the symmetry of the density histograms of the left and right hemisphere (after registration to a template), (2) definition of potential seeds, and (3) iterative optimization of level-set thresholds.

#### Manual segmentation of NCCT

The clinicians were instructed to manually segment on axial views, a slice of each of the five NCCT scans. Clinicians were provided the whole brain NCCT for review only. The segmentation was completed using the paintbrush mode in the ITK-SNAP software application.^1^

#### Manual segmentation of MRI

MRI-DWI images were manually segmented by trained personnel using ITK-SNAP to extract infarct lesions as reference. MRI-DWI lesions were registered to the NCCT images using Advanced Normalization Tools (ANTS) (16).

### Statistical analysis

The primary outcome of this study was assessed with the DICE similarity coefficient (DICE score). The DICE score measures the similarity of the lesion segmentations. It ranges from 0 to 1, when 0 represents no overlap and 1 represents perfect overlap. The DICE score is calculated by the following equation:


$$\mathrm{DICE}=\frac{\begin{array}{c} 2*\mathrm{Area}\;\mathrm{of}\;\mathrm{overlap}\;\mathrm{between}\;\mathrm{NCCT}\;\\ \mathrm{lesion}\;\mathrm{and}\;MRI\;DWI\;\mathrm{lesion} \end{array}}{\mathrm{Total}\;\mathrm{area}\;\mathrm{of}\;\mathrm{NCCT}\;\mathrm{and}\;MRI\;DWI}$$

In this study, the DICE scores were calculated for the following comparisons: (1) the AI-based HDT output and the registered MRI-DWI lesion, (2) the clinicians’ segmentation before viewing the AI-based HDT and the registered MRI-DWI lesion, and (3) the clinicians’ segmentation after viewing the AI-based HDT and the registered MRI-DWI lesion.

The DICE scores were summarized using mean and standard deviation (SD). To compare the difference of the DICE score for clinicians before and after AI-based HDT implementation, a three-level mixed-effects linear regression was performed with DICE score as the outcome, AI-based HDT implementation before versus after as the independent variable, and NCCT scans and clinicians as nested random effects.

Subgroup analyses were conducted on NCCT slices with large and small lesions, as well as NCCT slices segmented by consultants versus trainees.

All statistical analyses were performed with STATA 13.0 (Stata Corp, College Station, Texas, United States). *p*-values less than 0.05 were considered as indicative of statistical significance. Confidence intervals (CI) were set at 95%.

### Ethics

This study used data from the INSPIRE registry, a prospectively collected acute stroke clinical and imaging database. INSPIRE had central ethics approval by the Hunter New England Human Research Ethics Committee (HNEHREC 11/08/17/14.01), written informed consent was obtained for each patient for their data to be used as part of the INSPIRE registry. The INSPIRE registry and all associated analyses are conducted in accordance with the declaration of Helsinki.

## Results

### Data selection

A total of 32 clinicians participated in the study. Five clinicians were excluded as their drawings were incorrectly saved and unable to be read. For the remaining 27 clinicians, 26 clinicians had the 5 NCCT scan assessments completed before and after the AI-based HDT was provided, whereas 1 clinician had 2 NCCT scans reviewed and assessed. Thus, a total of 132 matched pairs of segmentations before/after AI-based HDT from 27 clinicians were included in this study.

### AI-based HDT performance against MRI-DWI gold standard

When compared to the true lesion reference on MRI-DWI, the AI-based HDT resulted in a mean DICE score of 0.62 (SD 0.05) in detecting the hypodensity region on NCCT slices. The AI-based HDT performance was consistent across the 5 NCCT slices, with DICE scores ranging from 0.54 to 0.66 (Table 2).

**Table 2:** Median DICE score across the 6 NCCT for clinicians and HDT.

Image ID	*N*	Clinician DICE before HDT implementation, mean (SD)	Clinician DICE after HDT implementation, mean (SD)	DICE of HDT
1	26	0.08 (0.18)	0.17 (0.22)	0.54
2	27	0.30 (0.32)	0.41 (0.28)	0.63
3	26	0.58 (0.21)	0.55 (0.20)	0.62
4	27	0.27 (0.32)	0.35 (0.30)	0.66
5	26	0.42 (0.24)	0.53 (0.17)	0.65
Overall	132	0.33 (0.31)	0.40 (0.27)	0.62

### Clinician performance against MRI-DWI gold standard

The overall mean DICE score for clinician interpretation of the NCCT slice before the AI-based HDT was applied was 0.33 (SD 0.31). The mean DICE score was 0.40 (SD 0.27) after clinicians viewed the AI-based HDT output (Table 2).

AI-based HDT use was associated with an increase of 0.07 (95% CI: 0.02–0.11, *p* = 0.003) in mean DICE score on mixed-effects linear regression, accounting for individual scans and clinicians as nested random effects.

**Figure 3:**
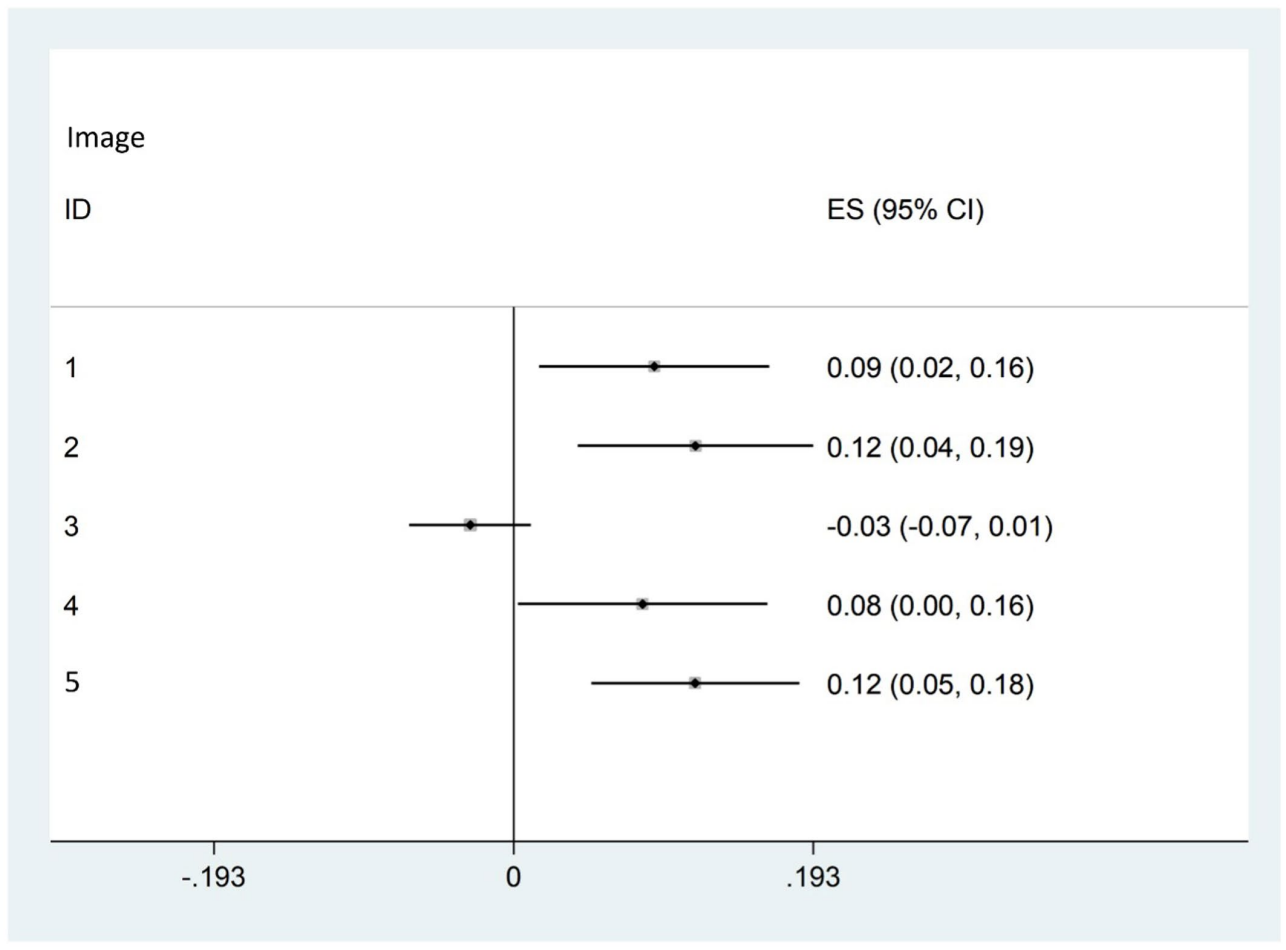
Forrest plot of DICE score changes before and after the HDT implementation.

For each NCCT, the performance of clinicians with and without the AI-based HDT in delineating NCCT lesion is summarized in Tables 2, 3 and Figure 3. The mixed-effects linear regression showed that DICE score was significantly increased in 4 NCCT scans after the AI-based HDT was reviewed, with the mean increase in DICE score of 0.09 (95% CI of 0.02 to 0.16) for Image ID 1, 0.12 (95% CI of 0.04 to 0.19) for Image ID 2, 0.08 (95% CI of 0.01 to 0.16) for Image ID 3, 0.07 (95% CI of 0.02 to 0.11) for Image ID 5. No such evidence of an increase was observed for Image ID 4, with the magnitude of −0.03 (95% CI of −0.07 to 0.01).

**Table 3:** Estimates of DICE change in mixed-effects linear regression.

Image ID	*N*	DICE changes, coefficient (95% CI)^a^	*p*-value
1	26	0.09 (0.02, 0.16)	0.017
2	27	0.12 (0.04, 0.19)	0.002
3	26	−0.03 (−0.07, 0.01)	0.162
4	27	0.08 (0.01, 0.16)	0.043
5	26	0.12 (0.05, 0.18)	0.01
Overall	132	0.07 (0.02, 0.11)	0.003

a Coefficient estimates the difference of DICE score before and after HDT implementation adjusting for the random effect of clinicians in mixed effects liner regression model.

### Performance of clinicians when assessing large versus small lesions

For the slices with large lesions (53 paired segmentations), the mean DICE score was 0.44 (SD 0.31) and 0.48 (SD 0.25) for clinicians before and after the AI-based HDT implementation, respectively. This improvement after AI-based HDT for the large lesion cases was not significant, with the mean increase in DICE score of 0.05 (95% CI: −0.02, 0.11, *p* = 0.192). For the slices with small lesions (79 paired segmentations), the mean DICE score was 0.25 (SD 0.29) and 0.35 (SD 0.28) for clinicians before, and after, the AI-based HDT implementation. The mean increase in DICE scores following AI-based HDT use was significant, 0.08 (95% CI: 0.02, 0.13, *p* = 0.004).

### Performance of consultant versus trainee

**Figure 4:**
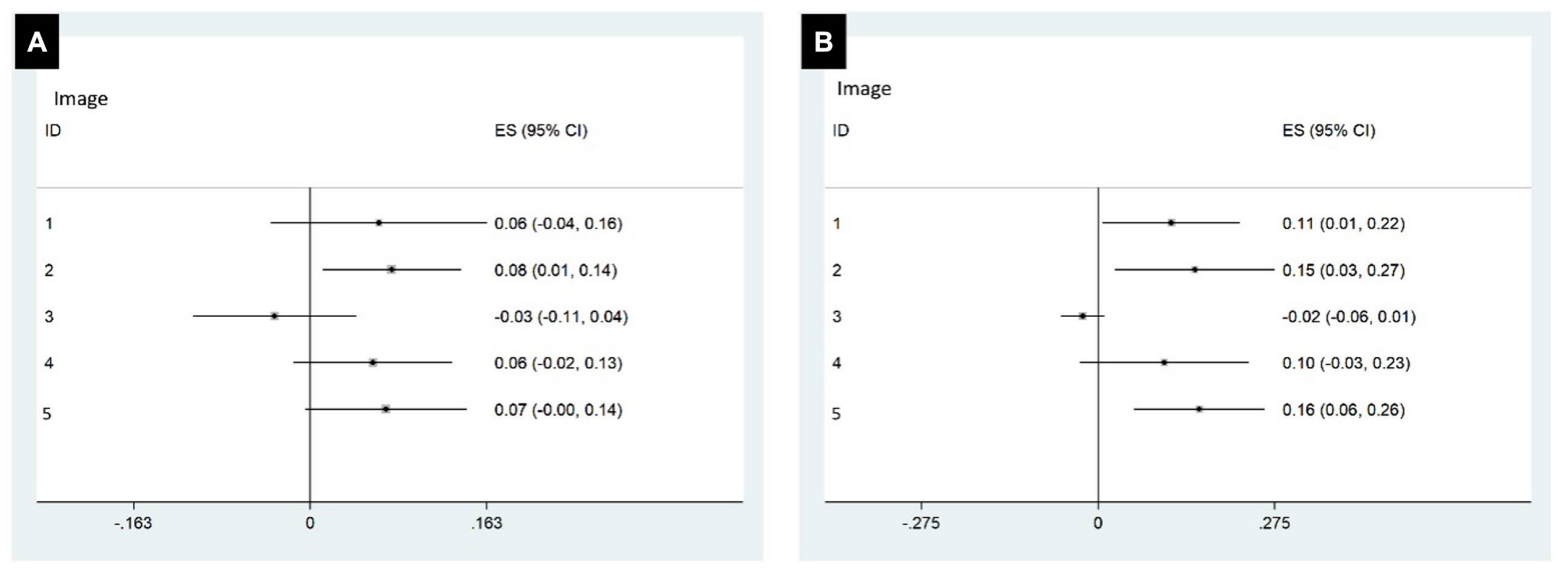
Forrest plot of DICE score changes before and after HDT implementation by consultants **(A)** and trainees **(B)**.

Among the 27 clinicians, 12 were consultant neurologists and 15 were trainee neurologists. For the subgroup of 12 consultants the mean DICE score was 0.40 (SD 0.33) and 0.45 (SD of 0.29) before and after the AI-based HDT implementation, respectively. This was not a significant improvement in DICE score for the consultants after AI-based HDT (mean difference in DICE = 0.04, 95% CI: −0.02, 0.107, *p* = 0.189). In contrast, the subgroup of 15 trainees produced a mean DICE score of 0.26 (SD of 0.28) before and 0.37 (SD of 0.26) after AI-based HDT, respectively, (Figure 3), demonstrating a significant AI-based HDT effect in improving DICE scores. The mean difference in DICE was 0.09 (95% CI: 0.03, 0.14, *p* = 0.004). The performance of consultants versus trainees for each scan is further illustrated in Figure 4.

### Success rate of HDT implementation

On review of the data, the clinicians decided not to change their original segmentations after reviewing the AI-based HDT output exactly half (50, 95% CI: 42, 58%) of the time. When we analysed only the segmentations that were changed after reviewing the AI-based HDT output, we noted the following changes to the original segmentation. For the consultants, mean DICE score was 0.42 (SD 0.31) and 0.51 (SD 0.19) respectively, and mean difference of DICE was 0.09 (95%CI: −0.01, 0.19) for the consultants. For the trainees, mean DICE was 0.31 (SD 0.29) and 0.51 (SD 0.16) respectively, and difference of mean DICE was 0.20 (0.12, 0.28).

## Discussion

We proved our primary hypothesis that the artificial intelligence (AI) based hypodensity detection tool (HDT) improved clinician detection of acute hypodensity on NCCT. Indeed, the AI-based HDT had relatively good performance in the detection of acute hypodensity on NCCT, and, it proved to be superior to all clinicians. Some might interpret our results as “AI should replace clinicians.” This is the first time the automated hypodensity detection algorithm has been used in clinical practise. The study was not designed to show superiority of the algorithm, it was designed to determine if it helped doctors’ identity hypodensity. A further study could be completed to show definite superiority of the algorithm.

Notably the AI-based HDT was most useful for neurology trainees, and when assessing small hypodense lesions. This is evident when looking at individual cases, given Image ID 3 had the best DICE score before AI-based HDT was applied. If all neurology trainees had changed their segmentation after reviewing the AI-based HDT, then the results may have been even more impressive.

These results suggest that clinicians applying AI-based HDT to acute stroke CT would have the greatest impact in regional, rural, and remote health care settings, where expert neurology consultants are not immediately available to assess acute stroke imaging for patients who need reperfusion therapy. Whilst any AI-based automated imaging tool does not replace the need for clinical judgment, AI-based HDT could provide support and guidance, and/or notify onsite doctors without expertise in NCCT assessment that the patient being assessed may have hypodensity on their NCCT, which can then help to influence treatment decisions. A further, larger validation study would also include radiology trainees, radiologist and neuroradiologists.

One of the most interesting findings of this study was the “resistance” of the doctors to change their assessment of the NCCT, even after reviewing the AI-based HDT output. Despite the clinicians knowing they were receiving an AI-based automated hypodensity detection output (which may be considered a source of bias), 50% of the participants did not change their original drawing. This assumes, 50% of the doctors were not willing to trust the AI-based hypodensity detection tool. This either speaks to the doctor’s (over)confidence in their ability to assess NCCT, or their wariness of relying on AI-based HDT to make an imaging decision (which has flow on effects on treatment decisions).

The main limitation of this study was the number of clinicians recruited and the resultant total of paired segmentations. The variability of results comes from the clinicians, with the scans used to test the clinician’s ability. The scans were selected to reflect different characteristics of lesions (size, location) to ensure the representative sample of scans, making them generalisable in acute stroke and a strength of this study.

In summary, these findings show the potential of AI-based HDT to significantly enhance clinician diagnostic precision and holds promise for its valuable application in clinical practice, particularly in neurology training and challenging diagnostic scenarios involving smaller hypodense lesions.

## Data availability statement

The raw data supporting the conclusions of this article will be made available by the authors, without undue reservation.

## Ethics statement

The studies involving humans were approved by Hunter New England Human Research Ethics Committee (HNEHREC 11/08/17/14.01). The studies were conducted in accordance with the local legislation and institutional requirements. The participants provided their written informed consent to participate in this study.

## Author contributions

AS: Conceptualization, Data curation, Formal analysis, Investigation, Methodology, Project administration, Validation, Writing – original draft, Writing – review & editing. MV: Conceptualization, Data curation, Formal analysis, Methodology, Writing – review & editing. LL: Conceptualization, Data curation, Formal analysis, Methodology, Writing – review & editing. AB: Methodology, Supervision, Writing – review & editing. LC: Methodology, Supervision, Writing – review & editing. MP: Methodology, Supervision, Writing – review & editing.

## Footnotes

^1^http://www.itksnap.org
